# Carbon nano-onions as fluorescent on/off modulated nanoprobes for diagnostics

**DOI:** 10.3762/bjnano.8.188

**Published:** 2017-09-07

**Authors:** Stefania Lettieri, Marta d’Amora, Adalberto Camisasca, Alberto Diaspro, Silvia Giordani

**Affiliations:** 1 Nano Carbon Materials, Istituto Italiano di Tecnologia (IIT), via Livorno 60, 10144, Turin, Italy; 2 Nanoscopy, Istituto Italiano di Tecnologia (IIT), via Morego 30, 16163, Genoa, Italy; 3 Department of Chemistry, University of Genoa, Via Dodecaneso 31, Genoa, 16145, Italy; 4 NIC@IIT, Istituto Italiano di Tecnologia (IIT), Via Morego 30, Genoa, 16163, Italy; 5 Department of Physics, University of Genoa, Via Dodecaneso 33, Genoa, 16145, Italy; 6 Department of Chemistry, University of Turin, via Giuria 7, 10125, Turin, Italy

**Keywords:** carbon nanomaterials, fluorescence, imaging, nanomedicine, nano-onion

## Abstract

Multishell fullerenes, known as carbon nano-onions (CNOs), have emerged as a platform for bioimaging because of their cell-penetration properties and minimal systemic toxicity. Here, we describe the covalent functionalization of CNOs with a π-extended distyryl-substituted boron dipyrromethene (BODIPY) dye with on/off modulated fluorescence emission activated by an acidic environment. The switching properties are linked to the photoinduced electron transfer (PET) characteristics of the dimethylamino functionalities attached to the BODIPY core. The on/off emission of the fluorescent CNOs is fast and reversible both in solution and in vitro, making this nanomaterial suitable as pH-dependent probes for diagnostic applications.

## Introduction

Nanomaterial-based probes (nano-probes) that are able to interact with disease markers or capable of sensing physiological changes in cells are widely used in diagnostic applications. In particular, fluorescent nano-probes are a relatively inexpensive platform compared to other biosensors and are capable of generating an optical output in response to a specific stimulus, making this technique operationally simple. Such stimuli can be a disease biomarker or changes in the cell chemical environment. In particular, intracellular pH plays a significant role in the physiological cellular activity indicating their health. Thus, a change in H^+^ can indicate physiological changes in the cells and tissues. Some of these events include cell proliferation and apoptosis [1], ion transport [2] and other cellular process and diseases such as cancer [3–5], Parkinson's, and Alzheimer's disease [6]. Despite the large number of nanotechnology platforms available to date for sensing applications [7], multishell fullerenes, known as carbon nano-onions (CNOs) [8–9], prepared by thermal annealing of detonation nanodiamonds (d-NDs) [10], are an attractive class of carbon nanomaterials (CNMs) for imaging, diagnostic and therapeutic applications, due to their unique properties. They exhibit low density and a high surface area to volume ratio [11–12] and have a spherical shape [13]. Moreover, they can be chemically functionalized, either covalently through chemical reaction [14] (e.g. oxidation [15–16]) or through adsorption of organic molecules by π–π stacking [17]. Our recent reports have shown that CNOs exhibit weak inflammatory potential and low cytotoxicity [16], and they are readily internalized by cancer cells and localize in the lysosomes [18–19]. Moreover, our in vivo studies performed on zebrafish (*Danio Rerio*) during the development stage demonstrated their biocompatibility [20]. We have previously shown that the pH-dependent switching ability of a dye is preserved when attached to CNOs [21] and on single-wall carbon nanotubes, [22] both in solution and in vitro*.* Thus, CNOs are suitable nanomaterials for biosensing applications. We exploited the photoinduced electron transfer (PET) and internal charge transfer (ICT) donor characteristics of the dimethylamino functionalities attached to a π-extended distyryl-substituted boron dipyrromethene (BODIPY) dye [23–24] to obtain a pH-sensitive nano-probe. Hence, CNOs grafted with BODIPY **3** molecules (fluo-CNOs) led to the development of a nanosensor which can be “turned on” in an acidic environment. Remarkably, the fluo-CNOs maintained the switching properties upon cell internalization, as they were “switched-on” in response to acidic pH. In vitro experiments on HeLa cells showed excellent cellular uptake and low toxicity of these fluorescent probes. Our findings pave the way for the development of fluorescent on/off modulated diagnostic nanomaterials.

## Results and Discussion

### Synthetic procedures

**Scheme 1:**
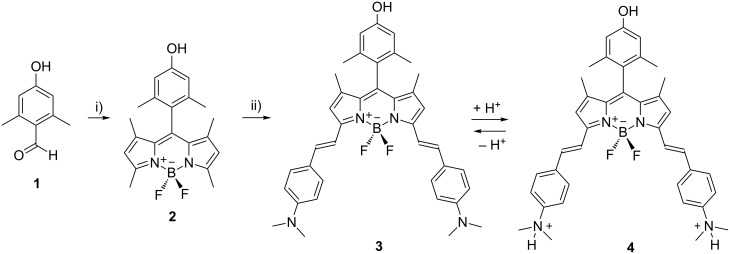
Synthesis of BODIPY derivatives **2** and **3**. i) 2,4-dimethylpyrrole, TFA, DCM, DIPEA, BF_3_OEt_2_; ii) 4-(*N*,*N*-dimethylamino)benzaldehyde, toluene, piperidine, glacial acetic acid, Mg(ClO_4_)_2_, Dean–Stark condenser.

**Scheme 2:**
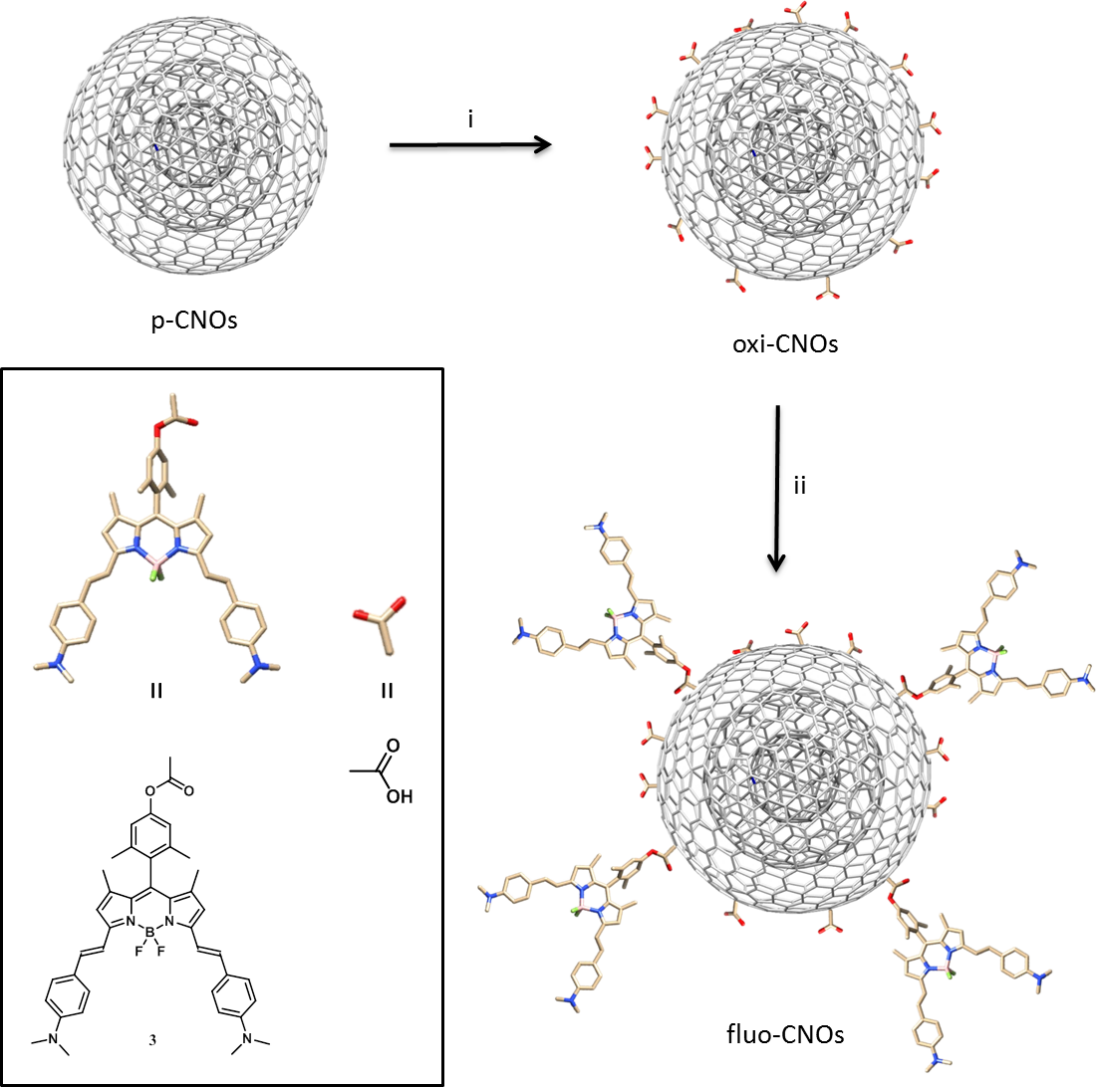
Procedure for the preparation of carboxy-functionalized oxi-CNO and fluorescently labelled fluo-CNO. i) Nitric acid, reflux, 48 h; ii) EDC, NHS, DMAP, **3**, dry DMF, N_2_, RT, 20 h.

The synthetic procedures are shown in Scheme 1 and Scheme 2. Compounds **1** and **2** were synthetized following a previously reported procedure [25–26]. The condensation with dimethylaminobenzaldehyde led to the NIR-BODIPY derivative **3**. The surface functionalization of 5 nm pristine CNOs (p-CNOs), synthetized by thermal annealing of d-NDs, was obtained by an oxidation process using a 3 M solution of nitric acid under reflux condition. The oxidation was performed directly on the sp^2^ carbon present on the p-CNOs surface, leading to the introduction of carboxylic acid groups. The highly functionalized oxidized CNOs (oxi-CNOs) were then grafted with BODIPY **3** molecules through an ester bond using 1-ethyl-3-(3-dimethylaminopropyl)carbodiimide (EDC) as the coupling agent at room temperature for 20 h (Scheme 2) to obtain fluo-CNOs.

### BODIPY

Compound **1** was synthetized from 3,5-dimethylphenol following a published procedure [25]. 3,5-dimethylphenol (46.5 g, 0.38 mol) and KOH (39 g, 0.70 mol) were dissolved in water (150 mL). CHCl_3_ (60 mL, 0.34 mol) was added drop wise with a dropping funnel and the reaction mixture was left to react for 24 h in total. The next day the brown reaction mixture was transferred in a separation funnel and the chloroform layer was separated from the aqueous one. The aqueous layer was then poured into a H_2_SO_4_ solution and a white precipitate formed. The precipitate was filtered and washed with fresh chloroform until the total removal of impurities was reached (4.5 g, 8%). ^1^H NMR (400 MHz, DMSO-*d*_6_) δ 2.49 (s, 6H), 6.52 (s, 2H), 10.28 (s, 1H), 10.32 (s, 1H). See Supporting Information File 1, Figure S1.

Compound **2** was synthetized according to a previously reported procedure [26]. 706 mg of 4-hydroxy-2,6-dimethylbenzaldehyde was dissolved in a degassed solution of EtOH (20 mL) and dichloromethane (DCM) (280 mL). 0.974 mL (9.4 mmol) of 2,4-dimethylpyrrole was added and the condensation was initiated with few drops of trifluoroacetic acid (TFA). The reaction mixture was stirred at RT for 16 h in the dark. Tetrachloro-1,4-benzoquinone (1144 mg/7.85 mmol) was added, followed by stirring for 30 min. The solvents were then removed under vacuum and the dark residue was redissolved in 150 mL of DCM. *N*,*N*-diisopropylethylamine (DIPEA) (4.9 mL) was added, and after 30 min, boron trifluoride diethyl etherate (BF_3_OEt_2_) (5.2 mL) was added. The mixture was stirred for 3 h. The crude was eluted on a silica plug using DCM before purification by column chromatography (SiO_2_, DCM/hexane 50:50, increasing amount of DCM) to obtain a red powder (850 mg, 49%). ^1^H NMR (400 MHz, chloroform-*d*) δ 1.43 (s, 6H), 2.08 (s, 6H), 2.56 (s, 6H), 4.78 (s, 1H), 5.97 (s, 2H), 6.63 (s, 2H). See Supporting Information File 1, Figure S2.

BODIPY **3** was synthesized by dissolving 220 mg of **2** (0.6 mmol) and 4-(*N*,*N*-dimethylamino)benzaldehyde (1.34 g, 0.009 mol) in 50 mL of dry toluene and deoxygenated by purging with di-nitrogen (N_2_). Piperidine (2.4 mL), glacial acetic acid (2.9 mL) and a catalytic amount of Mg(ClO_4_)_2_ were added and the reaction mixture was refluxed at 150 °C for 27 h with a Dean–Stark condenser. The crude was eluted on a silica plug using acetone before purification by chromatography (SiO_2_, EtOAc/hexane 2:8, increasing amount of EtOAc). The pure fractions were distilled, and the pure compound was precipitated from DCM in hexane to obtain a black powder (145 mg, 40%). ^1^H NMR (400 MHz, chloroform-*d*) δ 1.50 (s, 6H), 2.14 (s, 6H), 3.06 (s, 12H), 4.78 (s, 1H), 6.61 (s, 2H), 6.66 (s, 2H), 6.75 (d, *J* = 8.4 Hz, 4H), 7.21 (d, *J* = 16.1 Hz, 2H), 7.56 (d, *J* = 8.5 Hz, 5H), 7.61 (s, 3H). See Supporting Information File 1, Figure S3. ^13^C NMR (101 MHz, DMSO-*d*_6_) δ 158.04, 152.38, 151.44, 140.38, 137.26, 136.80, 135.86, 132.44, 129.11, 124.84, 124.50, 117.67, 115.33, 113.93, 112.67, 19.84, 13.65. See Supporting Information File 1, Figure S4. HRMS-ESI *m/z*: [M+H]^+^ calcd for C_39_H_41_N_4_OBF_2_, 630.3341; found, 630.3363.

### p-CNOs

The synthesis of small, pristine carbon nano-onions (p-CNOs) was performed by thermal annealing of detonation nanodiamonds (d-NDs) of 5 nm average particle diameter in a tube furnace under a positive pressure of helium at 1650 °C.

### oxi-CNOs

A dispersion of p-CNOs (50 mg) was prepared by ultrasonication (20 min at 37 kHz) in 30 mL of a 3 M solution of nitric acid (HNO_3_). The solution was stirred under reflux conditions for 48 h. The oxi-CNOs were separated from the reaction mixture by centrifugation (15 min at 1800 rpm) and filtered off on a nylon filter membrane (pore size 0.2 µm) and washed with dH_2_O, DMF, methanol and acetone. After drying overnight at RT, 51.2 mg of oxi-CNOs were obtained as a black powder.

### fluo-CNOs

A dispersion of oxi-CNOs (10 mg) was prepared by ultrasonication (30 min at 37 kHz) in 10 mL of anhydrous DMF. To the mixture 9.2 mg (0.08 mmol) of NHS, 12 mg (0.01 mmol) of DMAP and 14 µL of EDC were added consecutively. The reaction mixture was briefly sonicated and after the addition of 4 mg (0.0044 mmol) of BODIPY **3**, and the mixture was stirred at room temperature for 20 h under di-nitrogen atmosphere. The fluo-CNOs were filtered off thought a nylon membrane (pore size 0.2 µm) and washed with fresh DMF, THF and MeOH to remove the unreacted dye and the remaining reagents. 9.7 mg of fluo-CNOs were recovered as a black powder.

### PET and ICT effect

**Figure 1:**
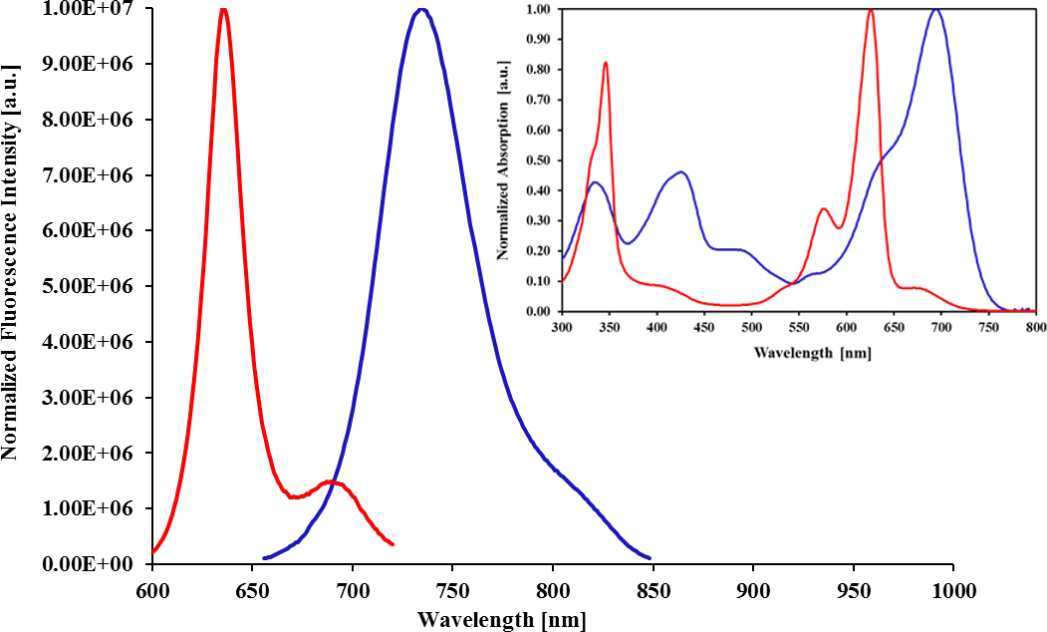
Emission spectra of BODIPY **3** (blue line: Excitation at 680 nm; emission at 737 nm) and BODIPY **4** (red line: Excitation at 600 nm; emission at 637 nm). Inset: absorption spectra of BODIPY **3** (blue line) and BODIPY **4** (red line) (solvent: chloroform). BODIPY **4** was protonated upon addition of H^+^ in the solution.

The on/off modulation of the fluo-CNOs emission is linked to the protonated/non-protonated form of the dimethylamino group attached to the BODIPY core. Upon protonation of the dimethylamino functional groups attached to the BODIPY core (BODIPY **4**, Scheme 1), which are capable of introducing photoinduced electron transfer (PET) [27] properties to the fluorophore, the dye molecule exhibited a bright red fluorescence with maximum emission centered at 637 nm in chloroform (Figure 1).

These PET groups were activated in neutral or basic environment (BODIPY **3**, Scheme 1), where the non-protonated form of BODIPY is present, and a low intensity maximum emission was observed at 737 nm in chloroform. They were instead deactivated upon protonation (BODIPY **4**) resulting in the enhancement of the fluorescence with emission maximum centered at 637 nm (see Figure 1, Table 1).

**Table 1:** Photophysical data for BODIPY **3** and **4**, absorption maximum (λ_abs_), and emission maximum (λ_em_).

Sample	Solvent	λ_exc_ (nm)	λ_em_ (nm)	λ_abs_ (nm)

BODIPY **3**	DMSO	680	768	712
BODIPY **3**	CHCl_3_	680	737	696
BODIPY **4**	CHCl_3_	600	637	624

**Figure 2:**
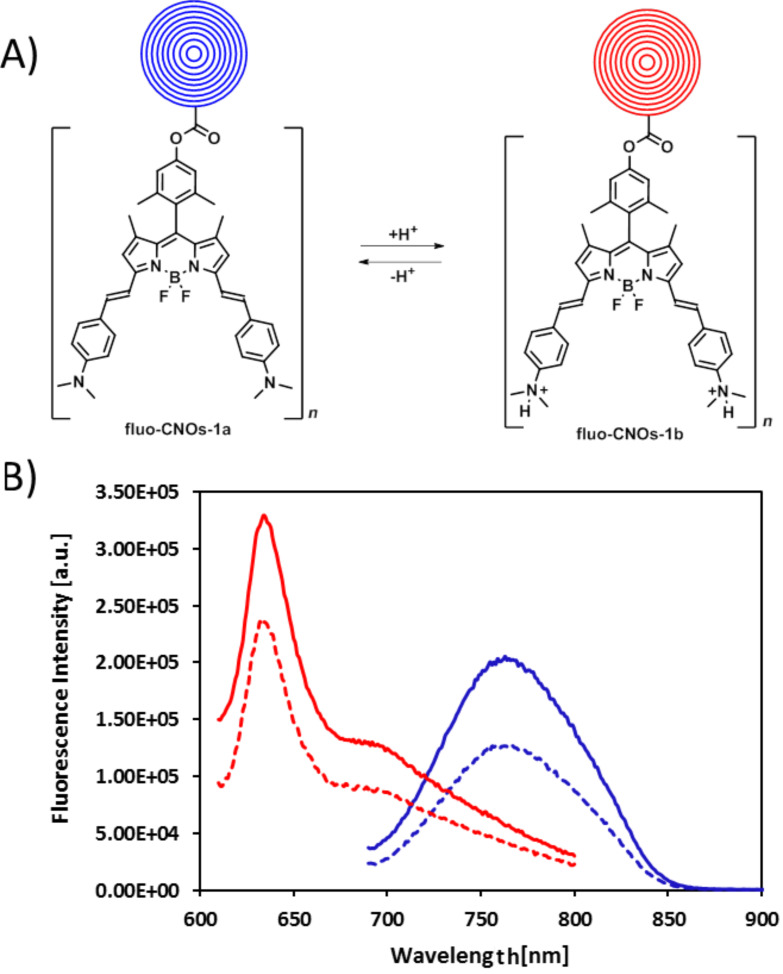
A) Protonated (fluo-CNOs-1a) and non-protonated (fluo-CNOs-1b) forms of fluo-CNOs. B) Emission spectra of fluo-CNOs-1a (blue line: Excitation at 680 nm; emission at 768 nm) and fluo-CNOs-1b (red line: Excitation at 600 nm; emission at 633 nm) in DMSO.

We observed that the on/off process is fast and reversible making this dye suitable for pH-dependent probes. Interestingly, this BODIPY sample also exhibited an internal charge transfer (ICT) characteristic resulting in a hypsochromic shift of the dye emission upon nitrogen protonation at acidic pH (Figure 1). At an excitation wavelength of 680 nm in chloroform, BODIPY **3** showed a maximum emission in the NIR window at 737 nm. Instead, when the dye was protonated (BODIPY **4**), a maximum emission at 637 nm was recorded when excited at 600 nm (Figure 1, Table 1). The ICT effect causes a variation of the intramolecular electron redistribution of the molecule. Accordingly, the amino groups of the dye attract electrons due to their electron-withdrawing characteristic, which led to an emission of BODIPY **3** at longer wavelengths (Table 1). This effect is reversed when the amino groups are protonated (BODIPY **4**). Despite the desired NIR window emission spectrum of BODIPY **3**, its quantum yield (Φ_F_) in DMSO is very low (Φ_F_ = 0.05), due to the active PET groups (amino groups), which causes a pH-dependent quenching of the fluorescent dyes. Overall, the non-protonated form of the dye (BODIPY **3**) is the switched-off form while the protonated form (BODIPY **4**) is the switched-on form. The successful functionalization of CNOs with BODIPY **3** was confirmed by photoemission studies. Upon photoexcitation at 680 nm, fluo-CNOs-1a (Figure 2) exhibited a maximum emission (λ_em_) at 768 nm, while fluo-CNOs-1b had a λ_em_ of 633 upon photoexcitation of 600 nm in DMSO. Hence, the ICT characteristics of the dye are preserved on the CNOs.

### Characterization of CNOs

**Figure 3:**
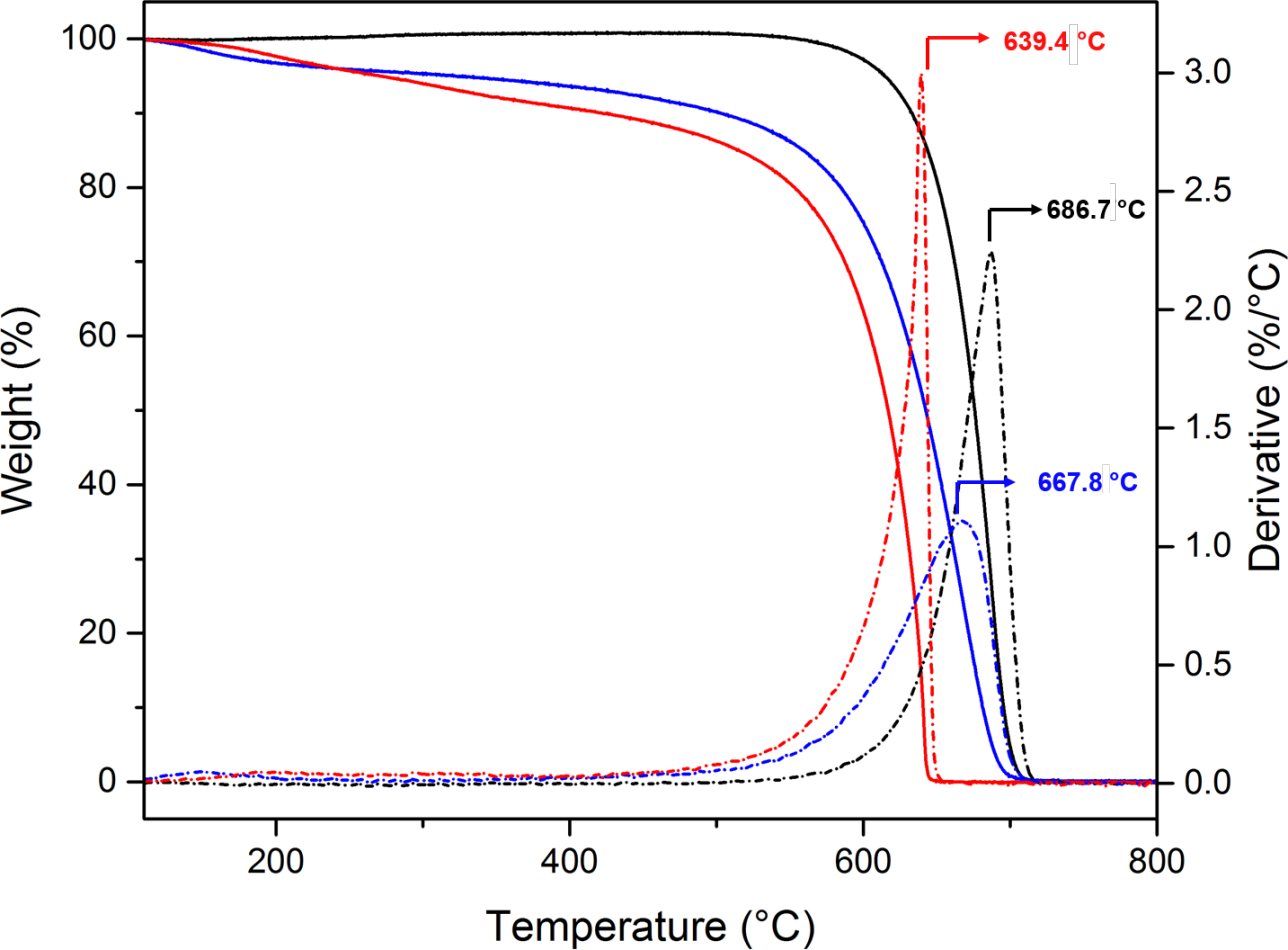
Thermogravimetric analysis (TGA) spectra of the functionalized CNOs. TGA (solid lines) and the corresponding weight loss derivatives (dotted lines) of p-CNOs (black), oxi-CNOs (blue) and fluo-CNOs (red). All experiments were run in air at a temperature rate of 10 °C min^−1^.

Oxi- and fluo-CNOs were characterized by thermogravimetric analysis (TGA) and Raman spectroscopy to prove the successful surface functionalization of the CNOs. The high degree of surface functionalization of the oxi-CNOs and the successful attachment of the dye molecules on the oxi-CNOs was observed by TGA analysis. The TGA spectrum of the oxi-CNOs performed in air (Figure 3, blue) showed a decrease in decomposition temperature from 686 °C to 668 °C, with a weight loss of 7.70% compared to the p-CNOs. From the TGA weight loss at 450 °C, a total of 122 carboxylic groups per CNO were estimated. The functionalization of oxi-CNOs with the dye molecules causes a further weight loss of the fluo-CNOs of 3.2% compared to the oxi-CNOs, with a decomposition temperature of 639 °C. We estimate that around four dye molecules per CNO were present. A high-resolution TEM image of the p-CNOs is given in Supporting Information File 1, Figure S5.

**Figure 4:**
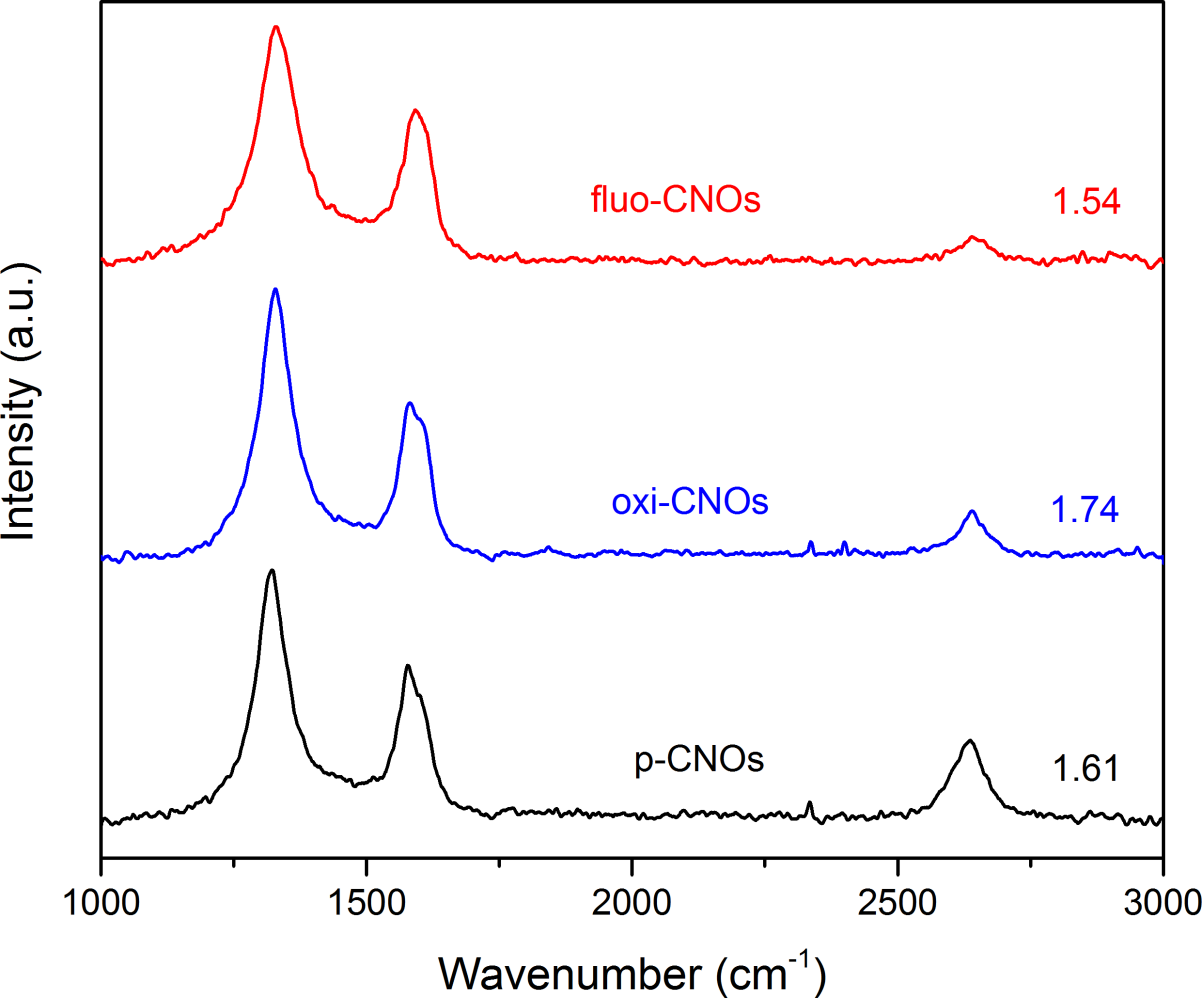
Raman spectra of the functionalized CNOs. The Raman spectra are normalized to the G-band at 1580 cm^−1^ and the ratios of the D-band to the G-band intensities are indicated.

The Raman spectra of p-, oxi- and fluo-CNOs showed the D-band (1320 cm^−1^) and the G-band (1580 cm^−1^) typical for CNOs [28]. The D-band at 1320 cm^−1^ refers to the defects present on the outer graphitic layer and is due to the presence of sp^3^-hybridized carbons. The G-band at around 1580 cm^−1^ corresponds to the E_2g_ mode of sp^2^-hybridized carbon frameworks. As shown in Figure 4, the D/G ratio increased in the oxi-CNOs compared to the p-CNOs due to the presence of defects on the CNO outer layer created by the oxidation process, confirming the introduction of carboxylic groups. Fluo-CNOs did not show any significant change in the ratio between the D and G bands, as the sample was excited with a built-in 632 nm laser which can alter the measurements due to background signal.

**Figure 5:**
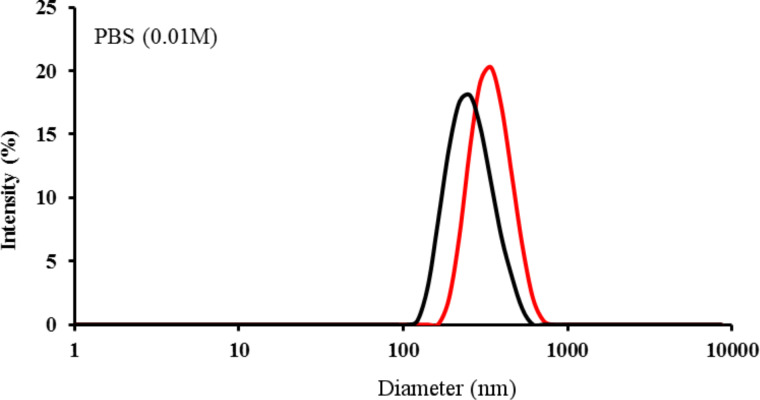
Effective hydrodynamic diameter of oxi-CNOs (black line) and fluo-CNOs (red line) in PBS at a concentration of 5 µg mL^−1^.

Dynamic light scattering (DLS) was carried out to determine the hydrodynamic radius of the dispersed CNOs (Table 2, Figure 5). DLS experiments were performed on CNOs samples dispersed in 0.01 M PBS to partially mimic the biological environment. The oxi-CNOs were found to have an effective hydrodynamic diameter of 274 ± 16 nm, while the fluo-CNOs have an average diameter of 357 ± 32 nm. The zeta potential changed from −45 ± 5 mV for the oxi-CNOs to −35.9 ± 1 mV for the fluo-CNOs, confirming the functionalization of the oxi-CNOs with the dye molecules (Table 2).

**Table 2:** Effective hydrodynamic diameter (0.01 M PBS) obtained from dynamic light scattering (DLS) measurements and zeta potential (phosphate buffer) of oxi-CNOs and fluo-CNOs at a concentration of 5 µg mL^−1^ in PBS.

Sample	Effective hydrodynamic diameter (nm)	Zeta potential (mV)

oxi-CNOs	274 ± 16	−45 ± 5 mV
fluo-CNOs	357 ± 32	−35.9 ± 1 mV

### Cytotoxicity studies

**Figure 6:**
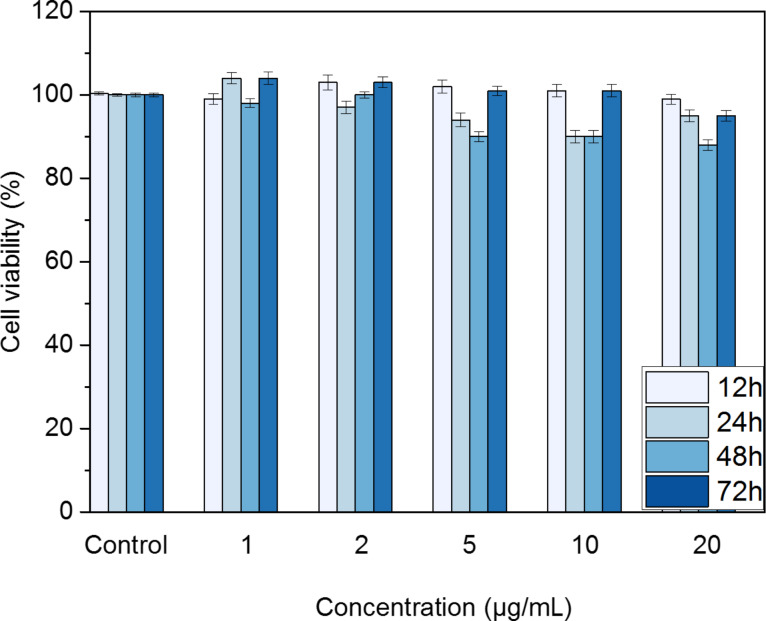
Cellular viability of HeLa cells treated with different concentrations (1, 2, 5, 10 and 20 µg mL^−1^) of fluo-CNOs for 12, 24, 28 and 72 h in DMEM (pH 7.4), revealed by the WST 1 assay. Cell viability (%) was evaluated for the CNO-treated samples against a non-treated control. As a positive control, the cells were incubated with 5% DMSO (showing a viability decrease of ca. 45%). Data are expressed as mean ± standard error as calculated from three separate experiments.

The possible adverse effects of fluo-CNOs on HeLa cells were tested by using a colorimetric assay (WST1). Cells were exposed to different concentrations of fluo-CNOs (1, 2, 5, 10 and 20 μg mL^−1^) for different time periods (12, 24, 48 and 72 h). Cells treated with only cell culture medium were used as a control. The cell viability percentage was above 80%, showing that CNOs exhibited moderate toxicity to the cells at the tested concentrations (Figure 6). The observed high viability of the HeLa cells treated with CNOs demonstrated their suitability for application as intracellular sensors.

### Confocal imaging

**Figure 7:**
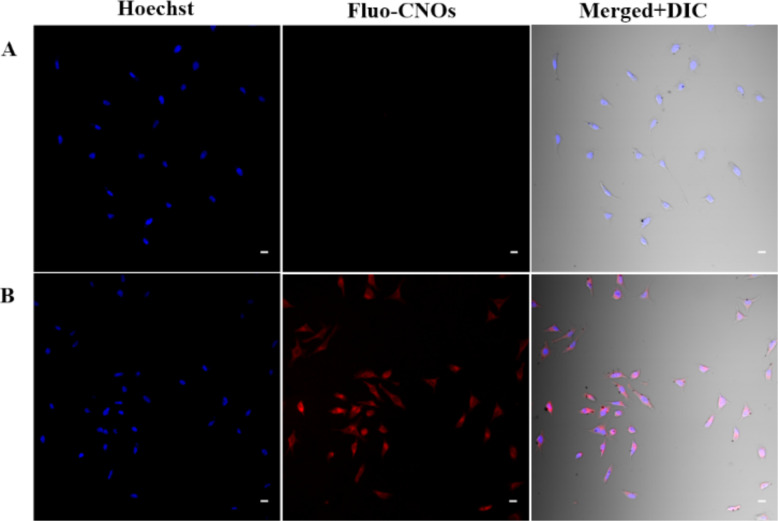
Confocal fluorescence images of HeLa cells treated with 20 μg mL^−1^ of fluo-CNOs. (A) PBS for 1 h, pH 7.4; (B) acid buffer for 1 h, pH 4.5; Scale bars = 20 μm.

Confocal imaging was performed on human cervical carcinoma (HeLa) cells treated with fluo-CNOs, in order to confirm the preservation of the PET and ICT characteristics of the dye attached to CNOs after cell internalization, hence the possible use of fluo-CNOs as pH-activated fluorescent probes. HeLa cells were incubated with fluo-CNOs (20 µg mL^−1^) and observed by confocal microscopy at different pH values in PBS to demonstrate the activation of the fluorescent emission in acidic environment. After the fixation, the nuclei were stained with a blue fluorescent dye (Hoechst 33232). Bright-field transmission images after fluo-CNO treatment confirmed that the HeLa cells were viable throughout all the experiments. From the fluorescence microscopy images (Figure 7), it was observed that cells treated with fluo-CNOs and maintained at a physiological pH (7.4) for 1 h exhibited no detectable red fluorescence signal (Figure 7A).

**Figure 8:**
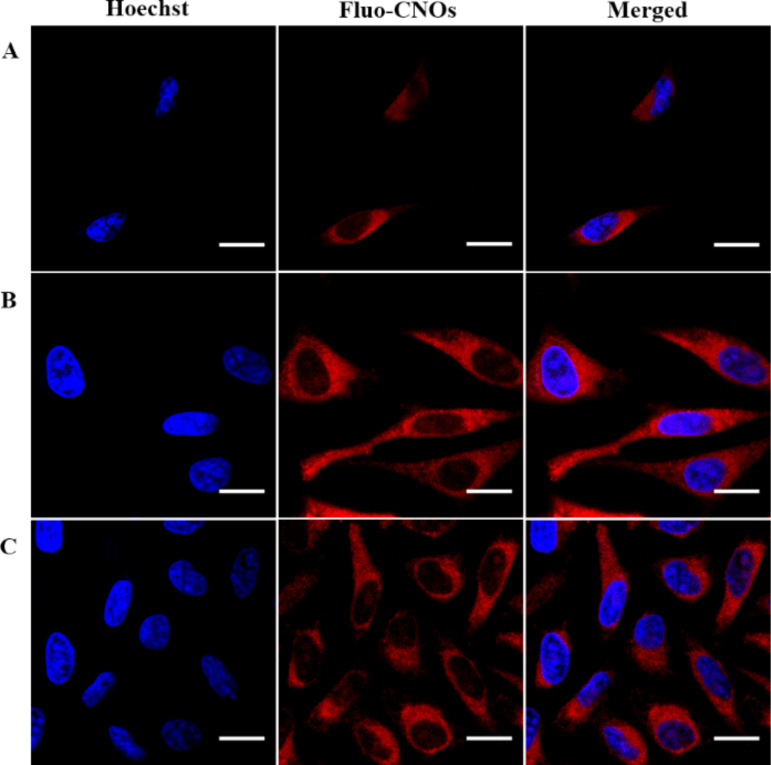
Cellular uptake and localization of fluo-CNOs in HeLa cells in acidic conditions (PBS, pH 4.5) observed by confocal fluorescence microscopy after incubation for 2 h (A), 12 h (B) and 48 h (C), respectively. Scale bars = 20 μm.

Successively, when the cells were incubated with acidic PBS (at pH 4.5), a strong red fluorescence signal was clearly observed in the intracellular region (Figure 7B). The overlay of fluorescence and bright-field images showed that fluo-CNOs were successfully internalized by cells and were distributed throughout the cytoplasm. Remarkably the cellular uptake of the fluo-CNOs was clearly observed soon after the incubation, as shown by the presence of CNOs inside the cell after 2 h (Figure 8A).

**Figure 9:**
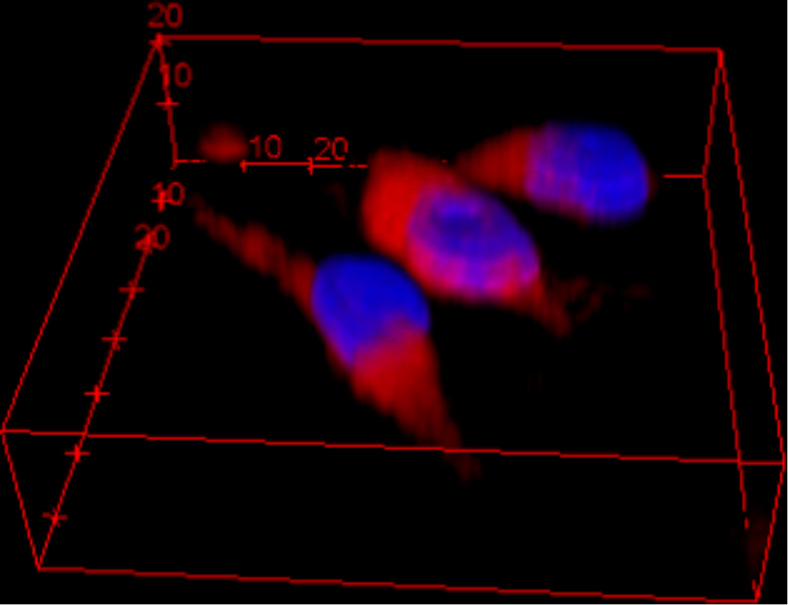
Three-dimensional reconstruction by confocal microscopy of cells incubated for 12 h with 20 µg mL^−1^ of fluo-CNOs in DMEM, and then for 1 h in acidic PBS (pH 4.5) and stained with Hoechst 33342.

After 12 and 48 h of incubation (Figure 8B,C), a progressive accumulation of fluo-CNOs was observed during the cell proliferation. Additionally, the efficient uptake was supported by a three dimensional reconstruction of cells treated with CNOs (Figure 9).

Finally, we demonstrated that the on/off fluorescent emission properties of the fluo-CNOs was reversible, as the intracellular fluorescence completely disappeared after treatment in a basic buffer (at pH 8.5) for 1 h (data not shown). Our results report that CNOs exhibit no fluorescence in neutral and basic environment (pH ≥ 7.0) and bright red fluorescence in acidic condition (pH ≤ 5.0). We proved that fluo-CNOs possess unique pH-switchable properties at acidic (pH 4.5), neutral (pH 7.4) and basic (pH 8.5) pH and can be potentially used as an intracellular pH-sensing nanoprobe.

## Conclusion

In summary, pH-sensitive BODIPY–CNO conjugates have been synthesized and characterized. The fluorescent carbon nano-onions (fluo-CNOs) were readily internalized by HeLa cells after 2 h of exposure and showed no major toxicity. The ability to switch the red fluorescence using pH control was demonstrated both in solution and in vitro, upon modification of the environmental pH, which resulted in an intracellular pH change. HeLa cells treated with fluo-CNOs exhibited no fluorescence at neutral and basic environment (≥7.0) and a bright red fluorescence in acidic condition (≤5.0). The on/off process was fast and reversible, making this nanomaterial suitable as a pH-sensitive probe for diagnostic applications.

## Experimental

### Materials

All solvents and reagents were purchased from Sigma-Aldrich in high purity grade. All reactions and measurements were carried out under ambient conditions, unless otherwise stated.

### Instrumentation

#### Thermogravimetric analysis (TGA)

TGA was conducted on a TA Q500 analyzer, using a platinum pan as sample holder. After equilibrating the sample at 30 °C for 5 min and then at 100 °C for additional 20 min, the measurement was performed in air using a heating rate of 10 °C/min. The sample weight was monitored until 900 °C.

#### Raman spectroscopy

Raman spectra were measured on a Horiba Jobin Yvon HR 800 UV LabRam Raman microscope. For the Raman measurements, the samples were deposited directly on a silicon wafer and excited with a built-in 632 nm laser.

#### Absorption and fluorescence spectroscopy

Absorption spectra were recorded on an Agilent Cary 8454 UV–vis diode array spectrophotometer. Fluorescence spectra were taken on a Horiba Jobin Yvon Fluoromax-4 spectrofluorometer in 1.00 × 1.00 cm quartz glass cells. The CNO samples were dispersed in DMSO to a final concentration of 1 mg mL^−1^. The dispersion of CNOs was sonicated for 15 min at 37 kHz and further diluted in DMSO to achieve final concentrations of 20, 10 and 5 μg mL^−1^.

#### Dynamic light scattering and zeta potential measurements

DLS measurements were performed on the Malvern Nano-ZS instrument operating in backscattering (173°) mode and analyzed with the Zetasizer software, with automatic selection of the optimal detector position and number of independent measurements. The CNO samples were dispersed to a final concentration of 1 mg mL^−1^ in PBS 0.01 M (PBS pH 7.4, composition 0.14 M NaCl, 0.0027 M KCl, 0.010 M PO_4_^3−^) The samples were sonicated for 10 min at 37 kHz and then diluted in 0.01 M PBS to achieve a final concentration of 5 μg mL^−1^. The CNO samples were sonicated for an additional 20 min and the particle size was measured. Zeta potential measurements were performed on the same apparatus using the disposable zeta potential cuvettes.

#### NMR spectroscopy

NMR spectroscopy was performed on a Bruker Avance III 400 MHz system (400.13 MHz for ^1^H and 100.62 MHz for ^13^C) in CDCl_3_ or DMSO-*d*_6_.

#### High-resolution mass spectrometry (HRMS)

The accurate mass measurements (HRMS) were performed on a Waters SYNAPT G2 high-resolution mass spectrometry instrument equipped with an electrospray ionization interface and coupled to a Waters ACQUITY UPLC. Electrospray ionization in positive mode was applied in the mass scan range 50–1200 Da. The analysis was performed on a Waters ACQUITY UPLC BEH C18 column 100 × 2.1 mm ID (particle size 1.7 µm) with an in-line filter. The mobile phase was 0.1% formic acid in H_2_O and 0.1% formic acid in acetonitrile.

#### Fluorescence quantum yield

Fluorescence quantum yields were determined by the comparative method published by Williams et al. [29]. The integrated fluorescence intensities of a known dye and the tested compound were compared and the fluorescence quantum yields were calculated using the following equation: 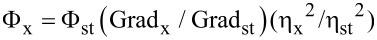
Φ_x_, where st and x denote the standard and test respectively, while Φ is the fluorescence quantum yield. Grad is the gradient obtained from the plot of integrated fluorescence intensity vs absorbance of the dye at the excitation wavelength. η represents the refractive index of the used solvents.

### Cell culture

HeLa cells (obtained from a human cervix carcinoma) were cultured in Dulbecco’s modified Eagle’s medium (DMEM) (Life Technologies) supplemented with 10% fetal bovine serum (FBS) (Life Technologies), 100 IU/mL penicillin and 100 μg mL^−1^ (Life Technologies) in humidified atmosphere at 37 °C with 5% CO_2_. HeLa cells were passaged at 80% confluency and split 1:10 in fresh media and discontinued after passage 15.

### Confocal imaging

Hela cells were plated on chambered coverglass (Thermo Scientific Nunc Lab-Tek II) and cultured overnight in the maintenance medium in humidified atmosphere at 37 °C with 5% CO_2_. Cells were incubated with 20 μg mL^−1^ of fluo-CNOs for 2, 5, 12, 24 and 48 h. As a control, the cells were left untreated (data not shown). After incubation, the cells were rinsed three times with phosphate buffered saline (PBS) (0.1 M, pH 7.4), fixed in a combination of paraformaldehyde (3%) and PBS and incubated with a solution of Hoechst 33342 (5 μg mL^−1^) (Sigma) for 20 min. Finally, the cells were rinsed three times and filled with PBS. Confocal fluorescence imaging was then carried out with a Nikon A1R laser scanning microscope and a plan apo 20× DIC M and a plan apo VC 60× oil DIC N2 objective. In order to switch the fluorescence on and off, the cells were incubated respectively with an acidic PBS solution (pH 4.5) and a basic PBS solution (pH 8.5) for 1 h before imaging.

### Viability assay

For the cytotoxicity assays, the cells were seeded at 5 × 10^4^ cells in in 96-well plates and incubated overnight at 37 °C in a 5% CO_2_ humidified environment. Fluo-CNOs were suspended in DMEM culture medium at final concentrations of 1, 2, 5, 10 and 20 μg mL^−1^, followed by sonication for 10 min at 37 kHz. The cells were exposed to the different concentrations of fluo-CNO for 12, 24, 48 and 72 h. As a positive control for cytotoxicity, the cells were incubated with 5% DMSO. Cell viability was determined using the cell proliferation reagent WST-1 (Roche Applied Sciences). After aspiration of the culture medium, a mixture of DMEM and WST1 reagent (1/10 volume) was added to each well. After 2 h of incubation at 37 °C with 5% CO_2_, the absorbance at 450 nm was measured in a standard plate reader (VICTOR3 V Multilabel Readers, PerkinElmer) (690 nm reference wavelength). The viability of cells in each well was determined as a relative percentage to the control well. Data were reported as a mean of three replicates and the error bars were the results of the standard deviations of these replicates.

## Supporting Information

File 1 Additional Experimental Data.
