# Supplementary material for: Carbon nano-onions as fluorescent on/off modulated nanoprobes for diagnostics
Source: Beilstein J Nanotechnol. 2017 Sep 7;8:1878–88. doi: 10.3762/bjnano.8.188 (PMC5629398; doi:10.3762/bjnano.8.188)
Supplement: File 1 — Additional Experimental Data. [file Beilstein_J_Nanotechnol-08-1878-s001.pdf]

Supporting Information

for

Carbon nano-onions as fluorescent on/off modulated nanoprobes for diagnostics

Stefania Lettieri[1], Marta d'Amora[2], Adalberto Camisasca[1,3], Alberto Diaspro[2,4,5] and Silvia Giordani*[1,6]

Address: [1]Nano Carbon Materials, Istituto Italiano di Tecnologia (IIT), via Livorno 60, 10144, Turin, Italy, [2]Nanoscopy, Istituto Italiano di Tecnologia (IIT), via Morego 30, 16163, Genoa, Italy, [3]Department of Chemistry, University of Genoa, Via Dodecaneso 31, Genoa, 16145, Italy, [4]NIC@IIT, Istituto Italiano di Tecnologia (IIT), Via Morego 30, Genoa, 16163, Italy, [5]Department of Physics, University of Genoa, Via Dodecaneso 33, Genoa, 16145, Italy and [6]Department of Chemistry, University of Turin, via Giuria 7, 10125, Turin, Italy

Email: Silvia Giordani - silvia.giordani@iit.it

* Corresponding author

Additional Experimental Data

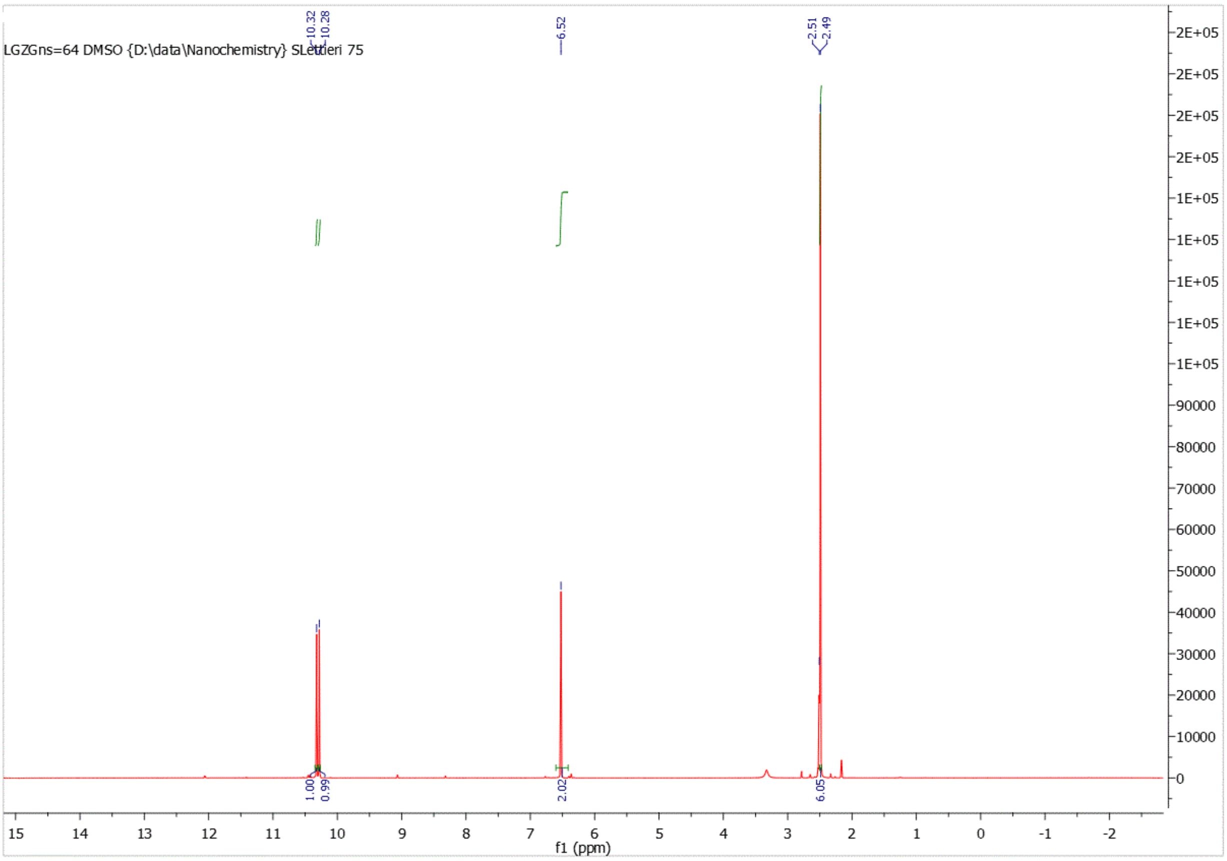

Figure S1: ^{1}H NMR spectrum in DMSO-d_6 of compound **1**.

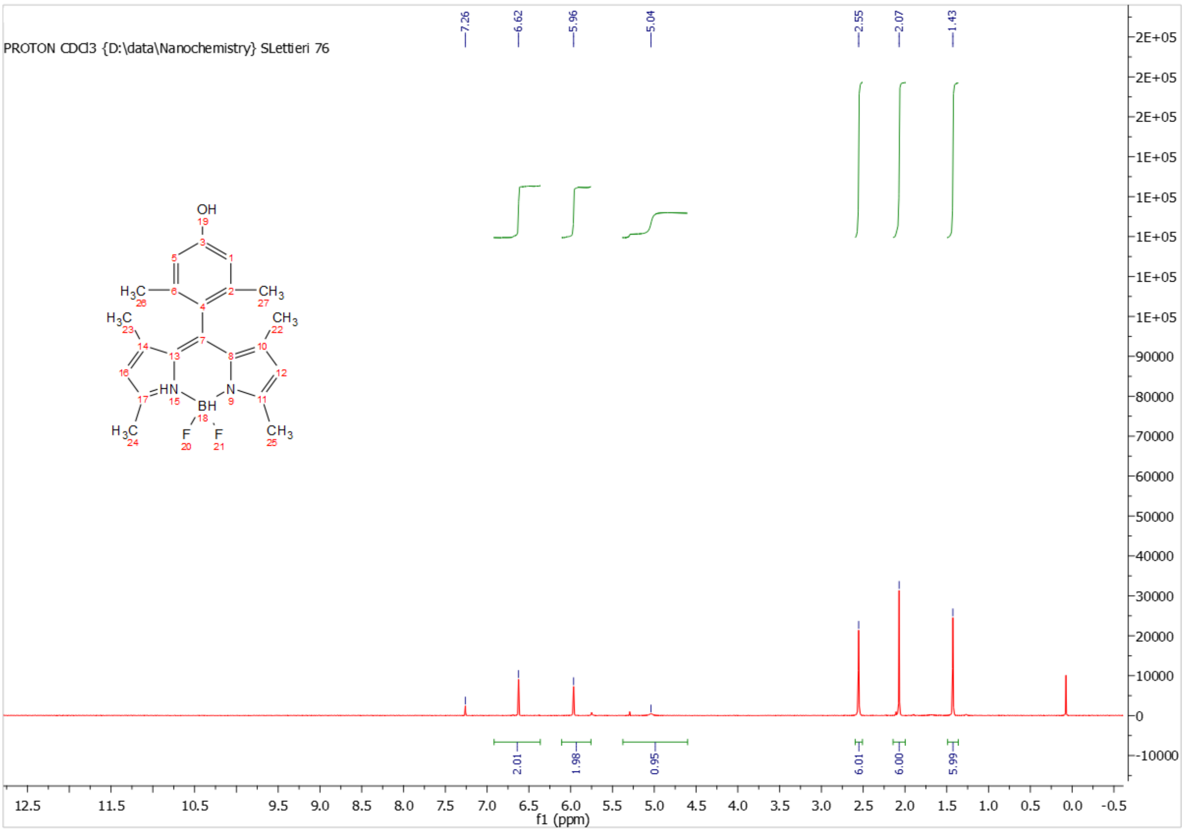

Figure S2: ^{1}H NMR spectrum in Chloroform-*d* of compound **2**.

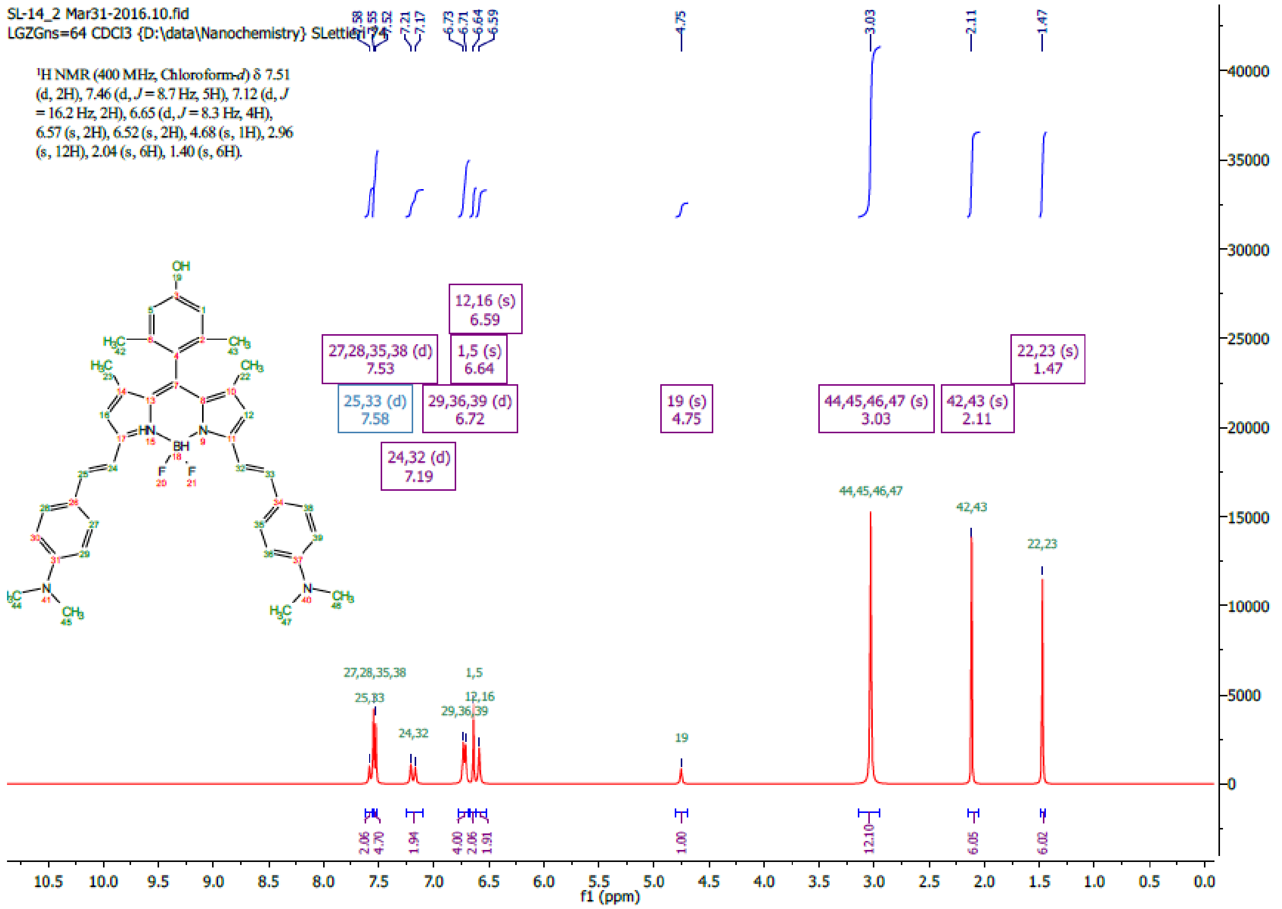

Figure S3: ^{1}H NMR spectrum in Chloroform-*d* of BODIPY **3.**

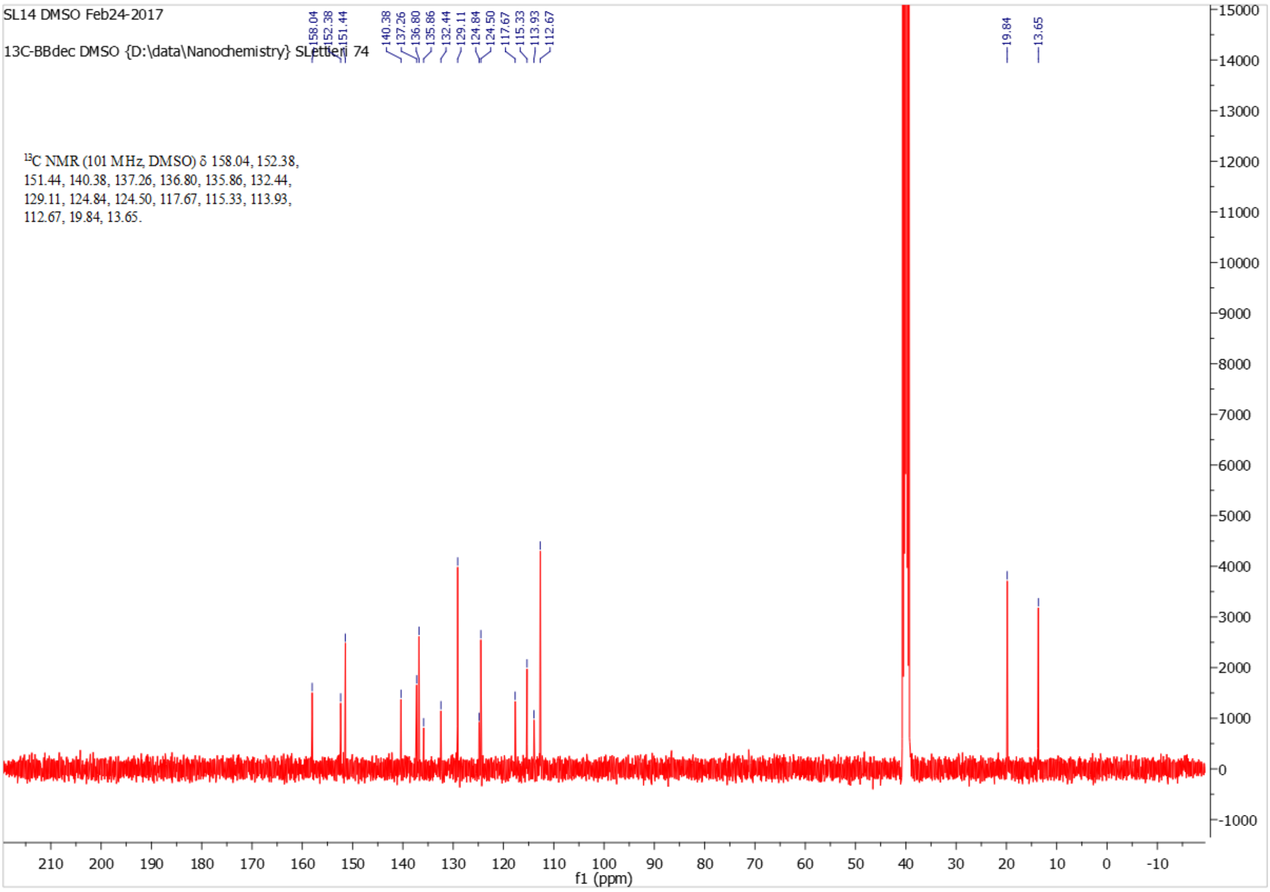

Figure S4: ^{13}C NMR spectrum in DMSO-d_6 of BODIPY **3**.

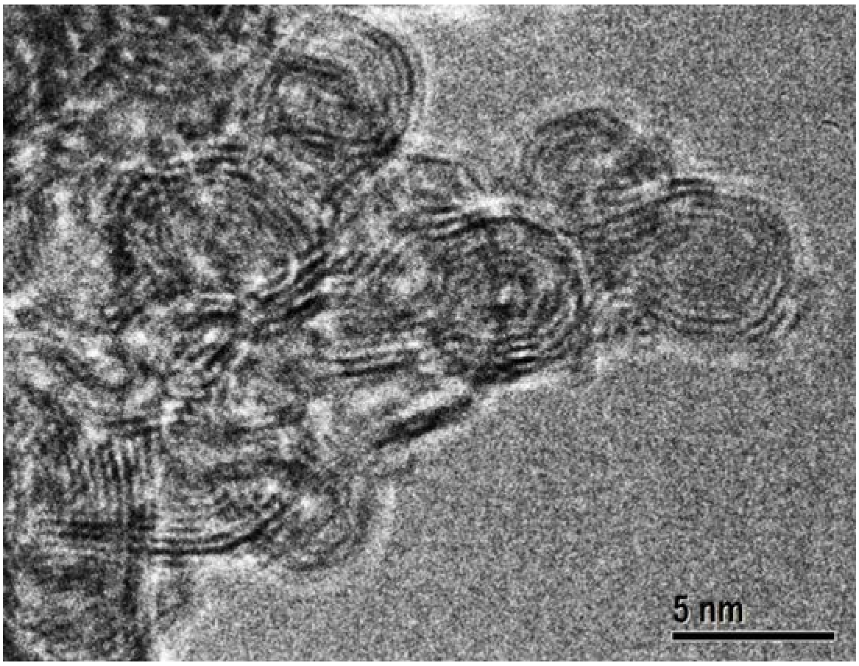

Figure S5: HR-TEM of p-CNOs.
